# Disasters, resilience, and the ASEAN integration

**DOI:** 10.3402/gha.v7.25134

**Published:** 2014-09-10

**Authors:** Don Eliseo Lucero-Prisno

**Affiliations:** 1 Department of Public Health, Xi'an Jiaotong-Liverpool University, Suzhou, China; 2 Faculty of Management and Development Studies, University of the Philippines (Open University), Los Baños, Philippines

The recent havoc caused by typhoon Haiyan in central Philippines provides an example of the grim reality of the impact of disasters in the Association of South-East Asian Nations (ASEAN) region. This is personal to me as many of my relatives and friends died and many more had their properties all washed away. You see, I was born in Tacloban and grew up in the city. It pained me to see my 87-year-old grandmother, a veteran of many typhoons, struggling to survive amid the stench of death and destruction. No doubt, the President of the Philippines was prompted to echo the need to strengthen the framework of cooperation in managing disasters among the ASEAN countries, being one of the best approaches to address urgent issues and to build resilience.

Needless to say, many efforts have already been initiated and continued in reducing the vulnerability of the region to the risk of disasters in the context of sustainable development. This includes the establishment of the ASEAN Committee on Disaster Management (ACDM) in 2003, which the ASEAN body elevated to a full-fledged committee. The ASEAN also promulgated the Agreement on Disaster Management and Response (AADMER) which is a legal instrument that binds all member countries in promoting regional cooperation and collaboration so as to lessen disaster losses and having a joint emergency response to disasters. This document is a manifestation of the commitment of the ASEAN to the Hyogo Framework for Action 2005–2015 supported by 168 countries (1).

Many observers, including the Secretary-General of the ASEAN, believe that the tipping point in the vigorous supranational policy approach on disaster management was instigated by the Indian Ocean tsunami disaster in 2004. The scale of the devastation of the tsunami was so massive that people, not only from the region, but even those from beyond, realized that disasters are realities that could strike anytime, anywhere. ASEAN's rhetoric was hinged on six Rs – reduce disaster risks, rebound quickly, reinvigorate leadership, renovate the plan, respond better, and revive the ASEAN's sense of community. Many of these narratives have been echoed time and time again. This echo gets louder as disaster strikes. What is thought provoking in this rhetoric is the idea of constant reflection; thus, the key terms – ‘renovate’, ‘reinvigorate’, ‘better response’, and ‘revive’. If there is a constant need for changing interventions and approaches, does this mean that previous actions have remained insufficient?

This is probably the main impetus to push the discussions in the ASEAN and go beyond the level of consensus building and move vigorously away from rhetoric and pronouncements. As urgent actions are required in disasters, readily available resources and decisions should be at the disposal at the supranational level. This beckons for the need to have a strong coordinating body that can easily deploy immediate interventions at any geographical location. A substantial amount of relief fund should be readily available for immediate disposal and disbursement anywhere. This would wean away the region from too much reliance on donors that normally arrive after the critical period of search and rescue phase and comes in with their own philosophies and approaches. A cooperative framework would definitely benefit the countries that need most help, which apparently are the countries most affected by disasters. A good framework is also imperative for a regional relief fund to make it substantially sufficient to be significant in delivering impact.

A supranational framework and body would, however, face many challenges, as ASEAN countries are diverse in many different ways. The ASEAN, however, is cognizant of the disparities in economic and financial capabilities of the countries necessary to build and sustain the activities in building disaster management capabilities. To bring the lesser-equipped countries on par with the others, the organization included in their 2009–2015 strategic framework the assistance of countries such as Cambodia, Laos, Myanmar, and Vietnam in enhancing their capabilities in disaster responses, and search and rescue by organizing training courses and workshop; provision of support through equipment and infrastructure for search and rescue and disaster responses; and providing more capacity building in disaster management and emergency response.

History has shown that time and time again, the ASEAN region will see more disasters. The region is prone to numerous hazards, big and small, that result in the accumulation of many losses of lives and properties. Its geographical location makes it vulnerable being placed in between two big oceans – the Pacific and Indian oceans – resulting in many typhoons, floods, landslides, and storm surges. Earthquakes, tsunamis, and volcanic eruptions are common occurrences as the region lies in between a number of tectonic plates. There are also forest fires and a number of much-publicized epidemics such as SARS, H1N1, and H9N7 that have caused havoc and hardships among the populations affected (2). MERS and the Ebola virus are a major scare as they pose threats to the region given the number of ASEAN migrant workers in the affected parts of the world.

According to the report of the ASEAN Disaster Risk Management Initiative (2), from 1970 to 2009 there were 1,211 reported disasters that comprised floods (36%), cyclonic storms (32%), earthquakes and tsunami (9%), epidemics (8%), landslides (7%), volcanic eruptions (4%), droughts (3%), and forest fires (1%). There were 414,927 deaths out of these reported disasters. The economic impact of all these disasters is so immense affecting the personal level to the regional level. There is also erosion of the health system, which renders governments unable to cope with the massive and quick rise of health needs of the population.

For example, the recent Haiyan disaster in the Philippines on 8 November 2013 resulted in more than 6,000 deaths, with 1,700 still missing, and 27,000 injured. The devastation has affected 14 million people, including some 5 million children. A total of 3.9 million people were forced from their homes. The United Nations and aid groups called to raise US$791 million to assist those affected (3). Reports show that the impact of the typhoon would have been minimized had there been better management of the risks prior, during and after the typhoon. Deaths were mostly due to the surge of water as people were caught unaware (4).

The ASEAN region also faces the challenges of ‘emerging’ disasters. These new ‘forms’ undoubtedly beg for a cooperative approach. For example, Malaysia Airlines Flight 370 may have directly affected few lives; however, its psychological impact on tourists and travelers was quite significant. The search for the plane and the people on board was an example why a multi-country effort was essential. Then Malaysia Airlines Flight 17 was another ‘global disaster’ that provided an impetus for a strong ASEAN stand. Albeit political, a unified voice of 10 countries with its innocent citizens killed can push swift actions at the international level. The same is true with political conflicts that resemble disaster scenarios such as the riots in Vietnam against Chinese nationals, the civil strife in Thailand, insurgency in southern Philippines, and the minority issues in Myanmar where political solutions become imperative.

Nuclear disasters such as Chernobyl and Fukushima may become a major issue in the future as countries such as Vietnam are planning four power reactors with the Philippines, Indonesia, Malaysia, Vietnam, and Thailand having research reactors (5). Obviously, a meltdown in China would directly affect the ASEAN. Oils spills, pollution, and poisoning of water systems from factories and mines, maritime piracy, and drug abuse should not be discounted.

Most of all, the impact of climate change should be seriously factored in as a transnational issue. As temperature increases sea level rises and drastic weather becomes more frequent resulting in more calamities (2). This trend is being observed in the region, which already showed massive effects on coastal populations, densely populated places, and the agricultural economy that populations are dependent on.

Yet, time and time again, we always see hope amid all this misery. Economies rebound and the populations recover despite the upheaval brought about by the havoc of calamities. Resilience is always a good story and is shown to be an enduring phenomenon. No matter how long the impact affects the populace, they become survivors and not victims; they pick up the pieces and move on with their lives. Yes, resilience is an area that is never focused on. Not even in the field of disaster research. This is why research becomes imperative and should be a major component of the approach in a unified disaster management framework. Documentation and research should be central in a supranational effort, as there is much learning that is yet to be discovered.

There are no arguments that counter the need for a strong and firm cooperative ASEAN effort in developing resilience against disasters – vulnerability is high, disasters are getting stronger and more frequent, and uniting small countries is prudent in pooling resources. An ASEAN disaster body with a strong mandate from the member governments in the context of an integrated ASEAN may be the impetus toward innovative and novel approaches in disaster preparedness and prevention and in cooperating to protect civilians. This might be the answer to the chaos that transpired during the Haiyan disaster in the Philippines. Hopefully, my grandmother would live to see the fruition of immediate and rapid responses during calamities. She already had enough with 87 years of disasters. And time is of the essence.

## References

[1] United Nations Office for Disaster Risk Reduction (2012). Synthesis report consultations on a post-2015 framework on disaster risk reduction (HFA2).

[2] United Nations Office for Disaster Risk Reduction (2010). Synthesis report on ten ASEAN countries disaster risk assessment.

[3] USAID (2013). Philippines – Typhoon Yolanda/Haiyan Fact Sheet No. 14.

[4] WHO (2013). Health response – Typhoon Haiyan (Yolanda).

[5] World Nuclear Association (2014). Asia's Nuclear Energy Growth. http://www.world-nuclear.org/info/country-profiles/others/asia-s-nuclear-energy-growth/.

